# Transcription regulation strategies in methylotrophs: progress and challenges

**DOI:** 10.1186/s40643-022-00614-3

**Published:** 2022-12-12

**Authors:** Xiaohan Huang, Qiaoqiao Song, Shuqi Guo, Qiang Fei

**Affiliations:** 1 School of Chemical Engineering and Technology, Xi’an Jiaotong University, Xi’an, 710049 China; 2 Shaanxi Key Laboratory of Energy Chemical Process Intensification, Xi’an Jiaotong University, Xi’an, 710049 China

**Keywords:** Methylotrophs, C1 bioconversion, Transcription regulation, Transcription factor, Promoter, CRISPR

## Abstract

As a promising industrial microorganism, methylotroph is capable of using methane or methanol as the sole carbon source natively, which has been utilized in the biosynthesis of various bioproducts. However, the relatively low efficiency of carbon conversion has become a limiting factor throughout the development of methanotrophic cell factories due to the unclear genetic background. To better highlight their advantages in methane or methanol-based biomanufacturing, some metabolic engineering strategies, including upstream transcription regulation projects, are being popularized in methylotrophs. In this review, several strategies of transcription regulations applied in methylotrophs are summarized and their applications are discussed and prospected.

## Introduction

Methylotrophs are a group of microorganisms that can reduce methyl-type one-carbon (C1) compounds, such as methane and methanol (Killham et al. 2007; Chistoserdova et al. 2009), which are potential substrates to produce biofuels and chemicals. Bioconversion of C1 molecules into macromolecular compounds by methylotrophs has aroused researchers' interest in terms of greenhouse gas reduction, biomanufacturing, and biological mechanism exploring (Hu et al. 2022). To date, many methanotrophic bacteria and methylotrophic yeasts belonging to methylotrophic classification (Baghban et al. 2019; Werten et al. 2019; Fabarius et al. 2021) remain unclear genetic background, leading to a block on the research of metabolic engineering reform.

It is known that an interpretation of gene behavior is particularly critical for expanding the industrial advantages of microbes. Regulation of transcription is the key to gene expression, which is a significant strategy applied in metabolic engineering aiming to excavate the biological metabolic mechanism of target compounds and make reasonable gene designs (Deng et al. 2021). Different transcription regulation strategies can be selected according to the diverse metabolic engineering requirements, which have been widely used in various model strains (Nielsen et al. 2016; Deng et al. 2022).

Establishing a library of gene transcription elements, such as promoters, is always a rational scheme to express heterologous genes (Cheng et al. 2022). A transcription factor (TF) engineering strategy is a feasible approach to obtaining a new gene-phenotype by binding specific DNA motifs to regulate gene expressions. Besides, CRISPR-mediated genome editing tools make in situ gene manipulation more convenient, especially CRISPR interference (CRISPRi) and CRISPR activation (CRISPRa) systems that have exhibited an indispensable role in the upstream reform of metabolic engineering owing to their accurate and efficient gene perturbation (Gilbert et al. 2014; Konermann et al. 2015; Peters et al. 2016). The strategies mentioned above are relatively mature and can meet most metabolic needs. However, more tools and approaches need to be further developed and optimized for C1-based cell factories due to a better understanding of methylotrophic biology.

Due to the global demand for carbon neutralization, it is necessary to develop biotechnology that can effectively use C1 compounds as raw materials to produce useful products for mankind. To enhance the advantages of methylotrophs in the fields of environment and biorefinery, the analysis of gene expression regulatory networks is particularly important. Generally speaking, transcription regulation is a significant strategy for the enhancement of microbial gene expression. RNA polymerase itself has no special affinity for the promoter and cannot be transcribed alone resulting in silent genes. Therefore, transcription requires many elements and auxiliary tools to fulfill various requirements. Thus, more genetic strategies are urgent to be exploited and modified for improving the industrial availability of methylotrophs from the perspective of transcriptional regulations. This paper is to summarize research progress and discuss applications of several important transcription regulation strategies based on methylotrophs.

## Promoter engineering

The promoter is a significant element to regulate the transcription initiation time and expression intensity at the transcription level. The promoter of glyceraldehyde 3-phosphate dehydrogenase P_*GAP*_ is the most frequently used constitutive promoter which was first identified in methylotrophic yeasts (Waterham et al. 1997, Killham et al. 2007). Even so, P_*GAP*_ is not always the optimal choice to meet the needs of metabolic engineering, so researchers continued to characterize other possible regions which have been summarized more recently. Several constitutive promoters have been further found and their abilities to regulate gene expression levels were ranked by green fluorescence intensity (Wetzel et al. 2016; Cai et al. 2021; Yan et al. 2022a). Interestingly, some promoters showed different magnitudes varied from cultural conditions. The P_*PDH*_ was stronger than P_*TRI*_ in glucose conditions, while the opposite result manifested under methanol culture (Yan et al. [Bibr CR78][Bibr CR79]) and P_*GCW14*_ seemed dominant in a glucose nutritional environment (Liang et al. 2013; Zhang et al. [Bibr CR83][Bibr CR84]). Overall, this is ubiquitous in methylotrophic yeasts, which is mostly caused by other regulators.

In methylotrophic bacteria, constitutive promoter P_*mxaF*_ of methanol dehydrogenase gene *mxaF* is known as strongest (Puri et al. 2015). Nevertheless, the applicability of this promoter seems not particularly broad especially when it is constructed on an expression vector, P_*mxaF*_ cannot show expectant activity (Garg et al. 2018a; Nguyen et al. 2018). This may imply the promoter may be strictly regulated by a specific element in the host *Methylomicrobium buryatense* 5GB1. In contrast, promoter P_*tac*_, whose strength is second only to P_*mxaF*_, is probably more suitable for transcription optimization of metabolic engineering in methanotrophs though it is heterologous (Amann et al. 1988). As reported previously, P_*tac*_ promoter was the most suitable for gene expression in *Methylomicrobium alcaliphilum* 20Z, leading to a double-fold gene expression upregulation of *budABD* gene cluster than its native promoter to produce 2,3-butanediol (Nguyen et al. 2018). With further research on methane metabolism, various promoters have been identified and tested in methane-utilizing strains as shown in Table 1. By comprehensive comparison, P_*tac*_ is the most generally used promoter for transcription strengthening of bioproduction.

**Table 1:** The intensity of different reported promoters in Methylotrophic bacteria

Promoter	Gene product	Condition	Strength	Host	References
P_*tac*_		Methane	Strong	*M. buryatense* 5GB1	(Puri et al. 2015)
P_*rpoD*_	Sigma 70 factor RpoD	Methane	Weak	*M. buryatense* 5GB1	
P_*lac*_		Methane	Weak	*M. buryatense* 5GB1	
P_*mxaF*_	Methanol dehydrogenase	Methane	Strong	*M. buryatense* 5GB1	
			Weak	*M. trichosporium* OB3b	(Lee et al. 2021)
P_*J23101*_		Methane	Weak	*M. buryatense* 5GB1	(Wilson et al. 2021)
P_*J23112*_		Methane	Inactive	*M. buryatense* 5GB1	
P_*J23117*_		Methane	Inactive	*M. buryatense* 5GB1	
P_*J23119*_		Methane	Strong	*M. buryatense* 5GB1	
P_*CT5*_		Methane	Strong	*M. buryatense* 5GB1	(Garg et al. 2018a)
P_*sMMO*_	Soluble methane monooxygenase	Methane	Strong	*M. silvestris* BL2	(Smirnova et al. 2018)
		Methanol	Weak	*M. silvestris* BL2	
		Acetate	Inactive	*M. silvestris* BL2	(Theisen et al. 2005)
P_*ectA*_	Ectoine	Low salinity	Weak	*M. alcaliphilum* 20Z	(Mustakhimov et al. 2010)
		High salinity	Strong	*M. alcaliphilum* 20Z	
P_*tal*_	Transaldolase		Strong	*Methylomonas* sp. DH-1	(Lee et al. 2021)
P_*DnaA*_	Chromosomal replication initiator protein		Weak	*Methylomonas* sp. DH-1	
P_*Integrase*_	Integrase		Weak	*Methylomonas* sp. DH-1	
P_*rpmB*_	50S ribosomal protein L28		Weak	*Methylomonas* sp. DH-1	
P_*hps*_	Hexulose 6-phosphate synthase	Methane	Strong	*Methylomonas* sp. strain 16a	(Ye et al. 2007)

Dynamic regulation of promoters is a lynchpin in transcription regulation and inducible promoters play a pivotal role throughout the strategy. P_*AOX1*_, P_*DAS*_, P_*FDH*_, P_*FLD*_, P_*TPS1,*_ and P_*SEO1*_ (Amuel et al. 2000; Park et al. 2007; Duan et al. 2018) have been gaining attention due to their inducibility. Most of them are mediated by methanol but P_*TPS1*_, which is driven by nearly 50℃ in *Hansenula polymorpha*. This may be a stress mechanism evolved to cope with high temperature. A similar P_*TPS1*_ promoter in psychrotolerant yeast *Guehomyces pullulans* was proved to be activated once the survival temperature fluctuates to maintain short-term cell homeostasis by synthesizing heat stress-related enzymes (Zhang et al. 2013a). Besides, a dynamic tetracycline promoter/operator system was constructed in *M. buryatense* for lactate biosynthesis and reached a maximum titer of 1.3 g/L (Henard et al. 2016). This tool contains a promoter P_*tetR*_ that only works on occasion with anhydrotetracycline, which will release the binding restriction of P_*tetR*_ and RNA polymerase by changing the conformation of the tetracycline repressor protein. A promoter derived from P_*tetR*_ was recently assembled to a 3-hydroxybutyrate expression vector in *Clostridium ljungdahlii* and the degree of gene downregulation under inducing and noninducing conditions was reflected by qRT-PCR data (Woolston et al. 2018).

The superior characteristic indicates that P_*tetR*_ will be an important candidate for the dynamic regulation of subsequent promoter engineering. Likewise, an expression system for monitoring the NADH: NAD^+^ ratio was set up in *Methylococcus capsulatus* (Bath) under the control of an arabinose-inducible promoter P_*BAD*_ (Ishikawa et al. 2017). As additives for dynamic regulation, arabinose holds less toxicity than anhydrotetracycline, resulting in a toxicity-free mechanism that needs to be coupled under the installation containing P_*tet*_, especially in the fermentation process with exacting requirements for high-density production.

To balance the core metabolic flux between the target products and by-products, selecting and optimizing an appropriate promoter is the only goal of the promoter engineering strategy. So far, diversified promoter libraries have been built to evaluate promoter candidates. The original method of building a library is to amplify the promoters of all genes and detect the interaction with proteins by 2D-PAGE, then further screen through fusing fluorescent proteins in vivo (Lee et al. 2021). However, the tedious process greatly increases the time cost in this way. To break this barrier, an Error-prone polymerase chain reaction (Ep-PCR) with the ability to obtain high-throughput mutation and inspection has been developed and is commonly used in methylotrophs (Blazeck et al. 2013). A P_*GAP*_ mutated library, from which mutagenic P_*GAP*_ with different activities from low to high could be selected, was constructed with Ep-PCR in *Pichia pastoris* (Qin et al. 2011) to adjust the preference of TFs for different mutated sequences of promoters (Nevoigt et al. 2006). Besides, promoter-predicted platforms will help select optimal promoter sequences, and hybridizations are contributed to building a mutated library (Vogl et al. 2018, Cazier et al. 2021). A computational framework was developed to predict promoter regions according to RNA-seq data sets in *M. buryatense* 5GB1 (Wilson et al. 2021). Given this, metabolic networks based on promoter regulation can be easily predicted and established.

As initiation elements in the translation process, promoter engineering strategies have been utilized for optimizing single or several genes to achieve reasonable improvements in metabolic levels. Five expression models driven by different synthetic promoter P_*ADH2*_ variants formed a small library in which SNT5 variant optimized by 2.2-fold compared with the original expression element has been screened out. This research indicates strong variants are powerful alternatives to the most widely used promoters in *P. pastoris* (Erden-Karaoglan et al. 2022). Garg accompanied with his colleagues optimized several heterologous metabolites producing pathways by examining the strength among a series of RBS variants and diverse promoters in *M. buryatense* 5GB1C, up to 70 mg/L of crotonic acid and 40 mg/L butyric acids, were obtained in engineered strain from methane (Garg et al. [Bibr CR26][Bibr CR27]). Nguyen et al. designed an RBS library calculated by a computational program to address the issue that overexpresses *E. coli*-derived constitutive lysine decarboxylase for cadaverine production from lysine in *Methylosinus trichosporium* OB3b, combining with the strong promoter P_*tac*_, 2.99 mg/L cadaverine could be obtained from methane, which is the first time to produce amino acids for feed and diamine compound for polyamides from methane using engineered methylotrophic bacteria (Nguyen et al. 2020).

## Transcription factor engineering

TF can target multiple binding sites owing to their similar or related functions, which is called the characteristic of global regulation, while one gene can also be modulated by several regulators (Spitz et al. 2012). Therefore, mining TFs and predicting binding motifs will facilitate further investigations of the nature of TFs themselves and the construction of transcription regulatory networks.

Since the similarity of the transcription regulatory networks between methylotrophic yeasts and *Saccharomyces cerevisiae*, TFs in methylotrophic yeast have been widely reported and reviewed (Ergun et al. 2021), so the research progress is much faster than that of methylotrophic bacteria. Due to the limited number of TFs identified in methylotrophic bacteria (Table 2), the selection of mining approaches falls in TFs research.

**Table 2:** Transcription factors reported in methylotrophic bacteria

Transcription factor	Gene annotation	Host	References
*watR*	Lactate-tolerant causal regulator	*Methylomonas* sp. DH-1	(Lee et al. 2019)
*ssrA*	tmRNA	*Methylomonas* sp. DH-1	(Nguyen et al. 2019)
*rnpB*	Ribozyme	*Methylomonas* sp. DH-1	
*nifH*	Nitrogen-fixing factor	*Methylacidiphilum* sp*. RTK17.1*	(Carere et al. 2019)
*nifD*	Nitrogen-fixing factor	*Methylacidiphilum* sp*. RTK17.1*	
*nifK*	Nitrogen-fixing factor	*Methylacidiphilum* sp*. RTK17.1*	
*nifE*	Nitrogen-fixing factor	*Methylacidiphilum* sp*. RTK17.1*	
*nifN*	Nitrogen-fixing factor	*Methylacidiphilum* sp*. RTK17.1*	
*nifX*	Nitrogen-fixing factor	*Methylacidiphilum* sp*. RTK17.1*	
*nifA*	Nitrogen-fixing factor	*Methylacidiphilum* sp*. RTK17.1*	
		*M. buryatense* 5GB1	(Guo et al. 2022)
*phoB*	Phosphate transport regulatory	*M. buryatense* 5GB1	(Hu et al. 2020)
*phoU*	Phosphate transport regulatory	*M. buryatense* 5GB1	
*rpoN*	Sigma-54 factor	*M. trichosporium* OB3b	(Stafford et al. 2003)
*ectR*	Ectoine biosynthesis gene repressor	*M. alcaliphilum* 20Z	(Cho et al. 2022)

According to the sequence conservation of the binding domain and regulatory domain, TFs can be identified from a series of differentially expressed genes in the transcriptome database, which has been widely applied in the metabolic analysis of methylotrophs. This database can be obtained by comparing the transcripts of different strains under the same growth condition. Lactate restrictive issue is a main limiting factor in biotransformation from methane to lactate. A lactate-tolerant causal regulator *watR* was screened from the transcriptome level between evolved strains and wild-type strains of *Methylomonas* sp. DH-1 (Lee et al. 2019), revealing the mechanism of acid tolerance and enabling further high-yield products. On the contrary, the same strains may show a discrepancy in transcription under different culture conditions. Global regulators *ssrA* and *rnpB* were identified by the transcriptome analysis under the cultural conditions of two carbon sources (methane and methanol), thus resolving the stress mechanisms under growth limitation and nutrient restriction in *Methylomonas* sp. DH-1 (Nguyen et al. 2019).

More recently, nitrogen fixation of methane-utilizing bacteria has received extensive attention. Researchers started with TFs to explore the mechanism, and then nitrogen-fixing factors *nifHDKENX* (Carere et al. 2019) and *nifA* (Guo et al. 2022) were found to be activated under carbon or oxygen-limiting conditions through transcriptome analysis; thus, methylotrophic bacteria could use nitrogen to synthesize glycogen for energy storage. This discovery may further trigger the thinking of carbon/nitrogen balance in methane-utilizing strains. Besides, some TFs that are not directly related to the target metabolic process can also be reflected from the transcription level due to the interconnected metabolic networks in methylotrophs. Phosphate transport regulatory cluster *phoBU* in *M. buryatense* 5GB1 was found to respond to the methane/oxygen ratio in the headspace, confirming an interaction between phosphate transport and carbon fixation (Hu et al. 2020). However, there are still some housekeeping TFs existing in methylotrophs that are unable to exhibit the obvious transcription undulation regardless of the growth conditions.

To break the aforementioned limitations, a total DNA sequencing comparison with those strains owning clear genetic backgrounds was carried out. A housekeeping sigma factor *rpoB* was mined in methylotrophs by gene homology analysis with *Escherichia coli* and this global TF was later applied in the identification of new strains (Madhaiyan et al. 2010; Paget 2015; Jia et al. 2020). Based on confirming binding sequences, the specifically regulated TFs can be targeted through the characteristic of DNA–protein interaction. Unfortunately, although this method has been applied in TFs exploring other strains (Pan et al. 2022), there are no relevant reports on methylotrophic bacteria so far.

**Fig. 1:**
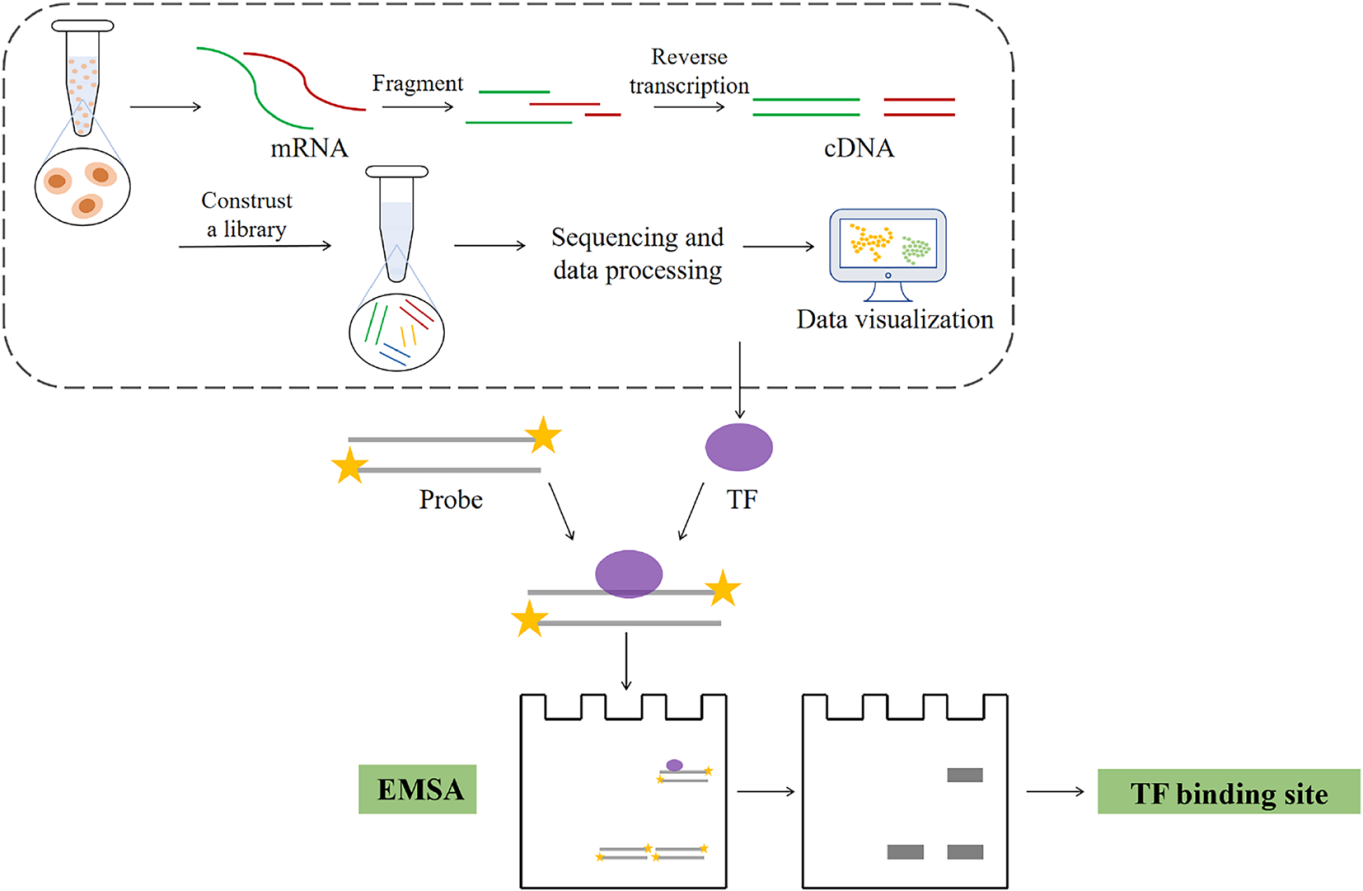
Mining strategy of TFs and identification of TFs binding sites

The DNA-binding motif is the key to clarify the regulatory role of TFs in metabolic pathways. Therefore, efforts have been made to identify transcription factor DNA-binding sites. Upstream DNA fragments will be amplified to contact with transcription regulators in vitro, and the DNA–protein interaction efficiency characterized by electrophoretic mobility shift assays (EMSA) will be used to evaluate whether there is a TF binding site in the fusion (Fig. 1). In methylotrophic yeasts, EMSA experimental evidence has helped explore the working rules of TFs. Ferroxidase Fet3 and permease Ftr1, involved in iron complexes formation, were both regulated by a suppressor-type factor PpFep1, which was proved to strongly bind with a specific 5’-(A/T)GATAA-3’ element. So that the coupled regulation between these two enzymes of PpFep1 can be further released by substituting or modifying one of the target regions (Miele et al. 2007). In *P. pastoris*, EMSA is also used to determine promoter regions in the meantime. Research showed that methanol expression regulator 1 (*mxr1*) could target the -141 to -138 region of glycerol transporter 1 (*gt1*) to run the expression manipulation. These four base pairs were just located on P_*GT1*_, which has been a known strong promoter in yeasts, inferring that *mxr1* may globally regulate all genes controlled by P_*GT1*_ (Zhan et al. 2017).

Transcription factor engineering has been applied for systematically regulation of metabolism in methylotrophs. In *P. pastoris*, Sun et al. found that co-expressing the transcription factor Hac1p and α-signal peptide-cutting protease Kex2p could increase the titer of recombinant lactoferrin from 121.6 μg/L to 35.6 mg/L, which has met the requirements of large-scale production (Sun et al. 2019). Liu et al. deleted an oxygen-related transcription factor Rox1p to obtain nearly double times improvement in β-mannanase enzymatic yield, realizing efficient production of animal feed additives from C1 compounds (Liu et al. 2021). Cho et al. recognized a MarR-like TF *ectR* in *M. alcaliphilum* 20Z, which was found to suppress the expression of the ectoine biosynthesis gene *ectD* by binding to the putative -10 sequence. Knocking out *ectR* could strengthen the transcription of *ectD* and ectoine production was enhanced 1.6-fold comparing the mutants to the original strains, thus solving the problem of transcription rate limiting in the conversion from methane to ectoine (Cho et al. 2022).

## CRISPR-based transcription regulation strategies

**Fig. 2:**
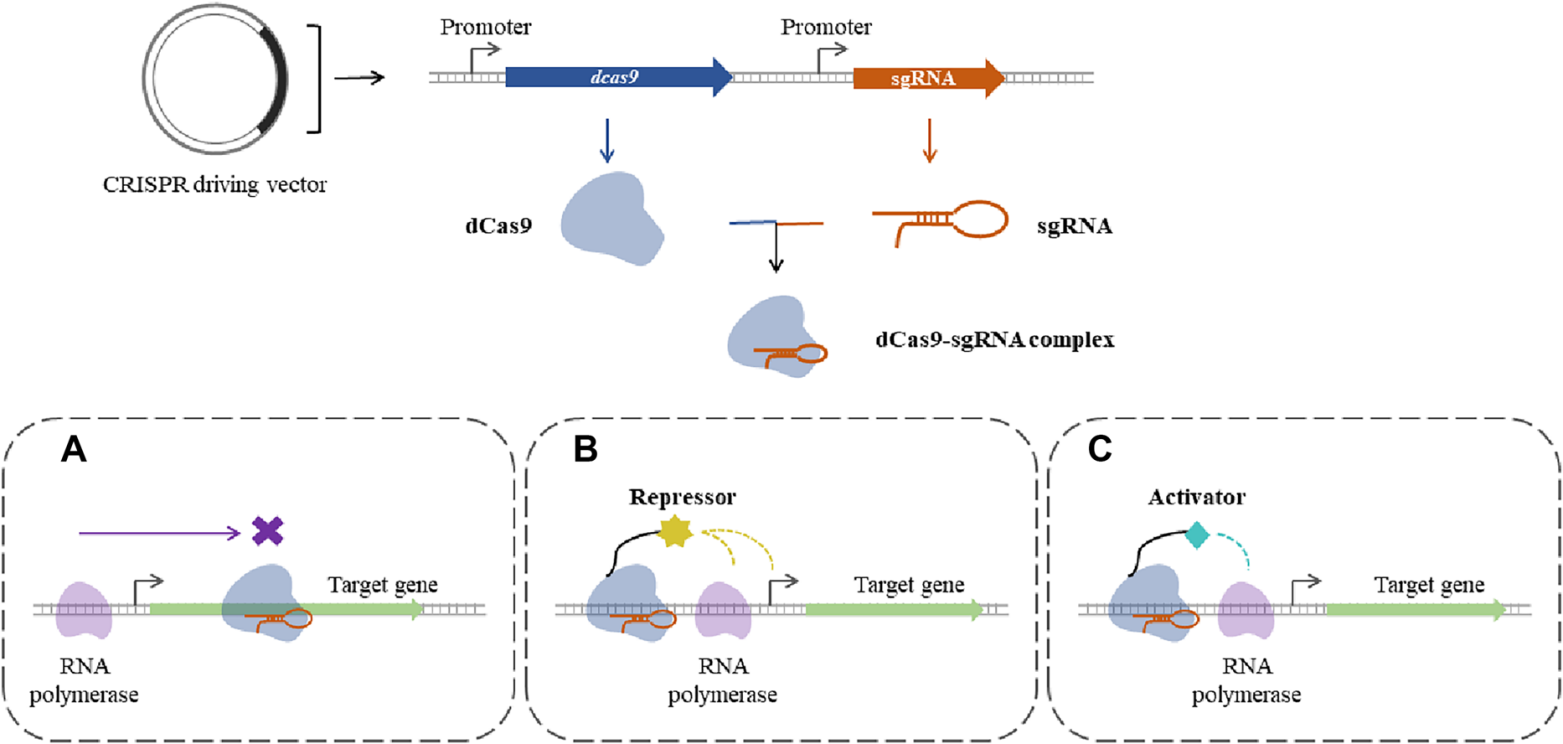
Transcription regulation mechanism of CRISPR-based strategies. **A** Regulation mechanism of CRISPRi: inhibit transcription process by blocking RNA polymerase; **B** Regulation mechanism of CRISPRi: repress the transcription of target genes by handling a repressor; **C** Regulation mechanism of CRISPRa: activate the transcription of target genes by handling an activator

With the development of clustered regularly interspaced short palindromic repeats (CRISPR) and CRISPR-associated (CRISPR-Cas) system, gene editing has become more unsophisticated (Czarnek et al. 2016; Peterson 2017). To its wide availability, CRISPR-Cas9 has turned out to be one of the most mainstream gene tools at present, only an endonuclease Cas9 protein and an artificial guide RNA (sgRNA) are needed. In terms of transcription regulation, CRISPR-mediated strategies without genetically altering have been exploited. On one hand, a deactivated Cas9 (dCas9), formed by inactivating Cas9 protein cleavage functional domain and retaining binding domain activity, is handled on sgRNA to catch hold of the target gene so that RNA polymerase cannot work normally due to the obstruction of dCas9-sgRNA complex in RNA polymerase binding or elongation leading to a noteworthy knockdown of the objective gene (as shown in Fig. 2A), that is called CRISPRi. On the other hand, by coupling transcription regulators and dCas9 protein, sgRNA is used as a guide to locate the upstream region of the gene to be manipulated; thus, the repressor or activator will exercise precise regulation (as shown in Fig. 2B, C), this is how CRISPRi and CRISPRa work (Schreiber-Agus et al. 1995; Fisher et al. 1996; Gilbert et al. 2013).

Despite a growing technology for transcription regulation, tools for gene inhibition and activation are still exploring in methylotrophs. CRISPRi and CRISPRa systems can change the transcription of interesting genes that have been widely used in the upstream process of metabolic engineering. Schultenkämper et al. first developed CRISPRi as a tool for gene repression in methylotrophic *Bacillus methanolicus* (Schultenkamper et al. 2019). They fused a *lac* repressor into the promoter of endogenous mannitol-1-phosphate 5-dehydrogenase gene *mtlD*, which might lead dCas9 toxicity in the host (Cui et al. 2018) to inhibit its activity, and the accessible CRISPRi system was assembled on an expression vector for further studies. This tool helped reveal the roles of regulatory gene *spo0A*, metabolic gene *mtlD,* and detoxification gene *katA* in *B. methanolicus*, as well as helped clarify the relationship between biofilm development and sporulation. Furthermore, based on the research above, a cooperative operation of two fructose-1,6-bisphosphate aldolase *fbaC*, *fbaP* was identified that the expression of these two genes is antagonistic (Schultenkamper et al. 2021).

All relevant studies serve to enrich the genetic background of methylotrophs. Mo et al. established another modified CRISPRi system in *Methylorubrum extorquens* AM1 considering the specificity of genetic elements. They amplified strong promoter P_*mxaF*_ to drive the sgRNA and chose an exogenous *dcas9* from *Streptococcus pyogenes* with lower GC content rather than the endogenous one. The optimal result showed that both *dcas9* and sgRNA were controlled by strong promoters only when would efficiency repression be obtained (Mo et al. 2020), which means there is a balance in the regulation of transcription elements. For solving this problem, a sequencing-based strategy for the selection of appropriate sgRNA in a specific host was presented and with huge potential to extend to many other microorganisms (Dalvie et al. 2020). However, an inevitable issue that the CRISPR-based system is easy to function on indispensable genes remains to bring off-target effect (Yang et al. 2018). Several strategies were summarized to deal with this effect (Manghwar et al. 2020), but in methylotrophs, more research is devoted to developing controlling strategies at the translational level to avoid the occurrence of an off-target effect (Zhu et al. 2021).

Although not all methylotrophic bacteria have developed advanced and efficient genetic manipulation tools, many of them perform the latent ability. For example, a CRISPR/Cas9 system was set up in *M. capsulatus* (Tapscott et al. 2019), indicating that the intracellular environment of *M. capsulatus* was compatible with CRISPR-based regulatory systems. Alternatively, class I CRISPR-Cas systems were described when comparing the complete genome sequence and taking homology analysis among *Candidatus Methylacidiphilum kamchatkense* Kan1, *Candidatus Methylacidiphilum fumariolicum* SolV, and V4 giving a lot of room for further development (Kruse et al. 2019).

## Prospective and challenges

Since methylotrophs play a pivotal role in C1 compound assimilation, the characteristics of strong robustness and less by-product make them receive great attention in biomanufacturing. The construction of transcription regulatory networks is conducive to the carbon flux rearrangement to meet the demands of metabolic engineering.

Although many intracellular regulatory promoters have been identified in methylotrophs, it is still urgent to find more elements to achieve the best combination for metabolic optimization. Compared with natural promoters, artificial ones can be freely designed in expression patterns and expression abundances according to different purposes to improve the accuracy of gene expression period, location, and condition (de Boer et al. 1983; Blazeck et al. 2012). Considering that many exogenous promoters are hard to reach the expected level in methylotrophs, a specific promoter regulation system in these strains is expected. Furthermore, artificial promoters could also retain the original promoter regulatory domain and reasonably fuse new regulatory sequences, which is a promising strategy in methylotrophs.

Obtaining target TFs and binding motifs is the basis of TF regulation studies. In previous reports, transcriptome analysis and EMSA are most frequently used in methylotrophic metabolic engineering, but these traditional mining methods are time consuming and labor intensive. To reach a higher efficiency screening, more high-throughput strategies are needed to be constructed in future research. Generally, the combination of substances must be accompanied by a change of energy. Isothermal Titration Calorimetry (ITC) is a thermodynamic technique to monitor any chemical reaction initiated by the addition of binding components which can recognize the interaction among DNA, protein, and other biological macromolecules (Du et al. 2016). DNA fragments of gene upstream regions can be set as probes to combine with candidate TFs before ITC analysis. All TFs binding results will be reflected from thermodynamic analysis in methylotrophs. The collection of ITC data can be further used to explore the laws of combination between TFs and binding regions and then to establish a computational database for accurate prediction.

In the aspect of transcription regulation tools, CRISPR-mediated strategies have been developed and applied in many microbial metabolic engineering (Zhang et al. 2016; Ishikawa et al. 2021; Ameruoso et al. 2022; Li et al. 2022). But in methylotrophs, few CRISPR-based tools have been constructed, which is mainly hindered by their genetic characteristics and loss of suitable dCas protein. Previous research showed an absence of the CRISPR/Cas system in wild-type *Methylocystis* sp. strain SC2 (Dam et al. 2013), which may lead to an incompatibility between heterologous CRISPR tools with the host. In addition, a restrictive modification system inhibits the direct transformation of the vector in *M. buryatense* 5GB1 (Yan et al. 2016); thus, CRISPR system based on plasmid construction is also affected. Therefore, constructing CRISPR-based platforms on the genome seems to be a universal solution. According to transcription regulatory features of the CRISPR system, dCas proteins from different sources can be pooled to form a library through codon optimization, where the core and adaptable dCas protein will be screened out. With the design of sgRNA, precise regulation of the whole genome can be achieved.

This review summarized three strategies applied in methylotrophs to facilitate research of metabolic engineering. At present, the progress on methylotrophs is striving to develop convenient and efficient approaches that can clarify the regulatory networks at the experimental level. Therefore, more works related to methylotrophs are required to establish tools for identifying global regulatory targets of specific transcription factors, methods for discovering all specific promoters and RBS, and platforms for optimizing efficient gene expression and modification.

## Data Availability

Not applicable.
